# Artificial Intelligence-Based Detection of Pneumonia in Chest Radiographs

**DOI:** 10.3390/diagnostics12061465

**Published:** 2022-06-14

**Authors:** Judith Becker, Josua A. Decker, Christoph Römmele, Maria Kahn, Helmut Messmann, Markus Wehler, Florian Schwarz, Thomas Kroencke, Christian Scheurig-Muenkler

**Affiliations:** 1 Department of Diagnostic and Interventional Radiology and Neuroradiology, University Hospital Augsburg, Stenglinstraße 2, 86156 Augsburg, Germany; judith.becker@uk-augsburg.de (J.B.); josua.decker@uk-augsburg.de (J.A.D.); florian.schwarz@uk-augsburg.de (F.S.); christian.scheurig@uk-augsburg.de (C.S.-M.); 2 Department of Gastroenterology, University Hospital Augsburg, Stenglinstraße 2, 86156 Augsburg, Germany; christoph.roemmele@uk-augsburg.de (C.R.); maria_kahn@gmx.de (M.K.); helmut.messmann@uk-augsburg.de (H.M.); 3 Department of Internal Medicine IV, University Hospital Augsburg, Stenglinstraße 2, 86156 Augsburg, Germany; markus.wehler@uk-augsburg.de; 4 Emergency Department, University Hospital Augsburg, Stenglinstraße 2, 86156 Augsburg, Germany

**Keywords:** chest radiograph, artificial intelligence, deep learning, early detection, COVID-19, pneumonia

## Abstract

Artificial intelligence is gaining increasing relevance in the field of radiology. This study retrospectively evaluates how a commercially available deep learning algorithm can detect pneumonia in chest radiographs (CR) in emergency departments. The chest radiographs of 948 patients with dyspnea between 3 February and 8 May 2020, as well as 15 October and 15 December 2020, were used. A deep learning algorithm was used to identify opacifications associated with pneumonia, and the performance was evaluated by using ROC analysis, sensitivity, specificity, PPV and NPV. Two radiologists assessed all enrolled images for pulmonal infection patterns as the reference standard. If consolidations or opacifications were present, the radiologists classified the pulmonal findings regarding a possible COVID-19 infection because of the ongoing pandemic. The AUROC value of the deep learning algorithm reached 0.923 when detecting pneumonia in chest radiographs with a sensitivity of 95.4%, specificity of 66.0%, PPV of 80.2% and NPV of 90.8%. The detection of COVID-19 pneumonia in CR by radiologists was achieved with a sensitivity of 50.6% and a specificity of 73%. The deep learning algorithm proved to be an excellent tool for detecting pneumonia in chest radiographs. Thus, the assessment of suspicious chest radiographs can be purposefully supported, shortening the turnaround time for reporting relevant findings and aiding early triage.

## 1. Introduction

The AWMF (Guidance Manual and Rules for Guideline Development) recommends chest radiographs (CRs) for patients clinically suspected of community-acquired or hospital-acquired pneumonia [1]. As a result, patients with dyspnea or presenting other respiratory symptoms for pneumonia in the emergency department are usually given a CR. CRs have the advantage of lower radiation exposure, faster feasibility and better equipment portability compared to other imaging modalities, e.g., computed tomography (CT) [2,3]. This diagnostic examination can provide supplemental and timely information regarding a patient’s cardiopulmonary condition and probable changes (acute and chronic) from COVID-19 infection [4,5]. Additionally, the ongoing COVID-19 pandemic has been challenging healthcare systems all around the world since December 2019. Accordingly, the number of CRs performed is increasing. In the context of high levels of infection and an increasing number of variants of concern, the early detection and isolation of patients is very important. This especially challenges radiology departments. Studies have shown that with faster reporting of pneumonia in CRs, the median length of hospital stays is significantly shorter, the likelihood of receiving appropriate therapy is higher, and the probability of infectious spread is lower [6,7].

However, the interpretation of CR examinations is variable and examiner-dependent [8,9]. To increase the sensitivity and specificity of imaging patterns for pneumonia in CR, deep learning (DL) algorithms must become more prevalent. Prior studies have shown that the use of artificial intelligence (AI) significantly improves the detection of pneumonia in CR [10,11,12,13,14,15,16,17,18,19]. However, the number of relevant studies is comparatively low [11,16].

Given the large number of examinations, reporting using AI can highlight CRs with abnormalities, helping to prioritize reporting by radiologists. Further, where CRs are initially evaluated by clinicians outside regular operations, AI can be of assistance. In this situation, a well-functioning evaluation of CR by AI can significantly support clinicians’ decision making.

This retrospective study evaluates the accuracy of a commercially available DL algorithm in detecting any kind of pneumonia in CR, aiming to prove its reliability and robustness in a large real-world patient collective during the COVID-19 pandemic in Germany. In addition, the assumed specific imaging patterns of COVID-19 and non-COVID-19 pneumonias are contrasted according to an evaluation performed by experienced radiologists and evaluated for their predictive value.

## 2. Materials and Methods

The conduction of this retrospective study was approved by the institutional review board (“Beratungskommission für klinische Forschung—BKF”, Augsburg; ID: BKF2020-28). All data were fully anonymized. The ethics committee waived the requirement for informed consent. 

### 2.1. Patient Collective

This study includes a total of 948 patients of legal age who presented with respiratory symptoms raising the suspicion of a pulmonal infection necessitating hospitalization. All patients received a CR on admission directly in the emergency department or after referral from external clinics and practices. This study includes data from the first and second waves of the COVID-19 pandemic. First-wave data recording began with the first patient testing positive in our hospital on 3 February 2020 and lasted until 8 May 2020 (*n* = 321 patients). The second wave started on 15 October 2020 and included all patients until 15 December 2020 (*n* = 627). Sex, age, confirmed COVID-19 infection by repeated RT-PCR and, if present, data about other pulmonary pathogens were included in the data collection. All other pulmonary pathogens were summarized as non-COVID-19 infections and are not investigated in more detail.

### 2.2. Imaging Evaluation

**Figure 1:**
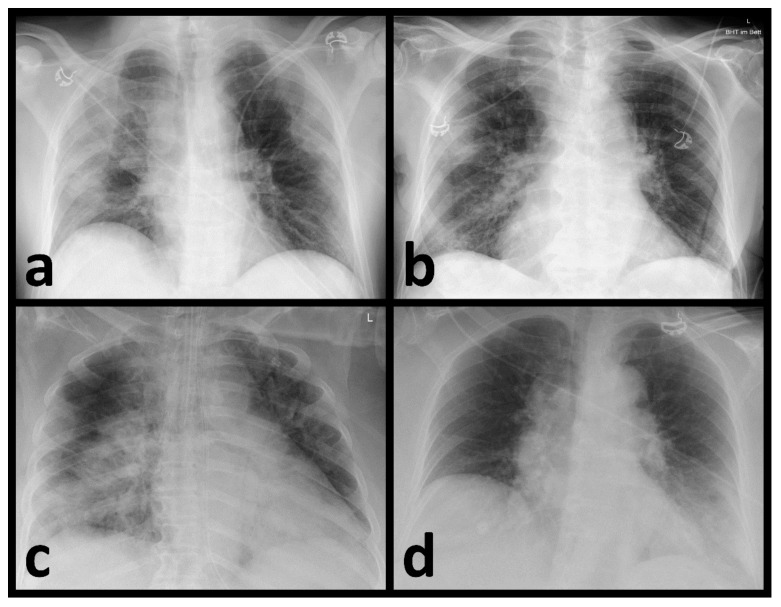
Chest radiographs with the distinguished distribution patterns regarding the probability of COVID-19 infection. (**a**) Typical (bilateral, peripheral opacifications), (**b**) almost typical (unilateral, peripheral opacifications), (**c**) non-typical (limited to one pulmonary lobe consistent with a lobar pneumonia) and (**d**) indeterminate (opacifications that could not be clearly classified as typical, almost typical, or non-typical).

All CRs were digitally recorded and conducted in compliance with the applicable regional statutory requirements. For this study, only the first CR on admission was included. All available projections were used. The retrospective assessment of the radiographs by the radiologists and the AI program were carried out separately and independently of each other. A senior resident and an experienced senior radiologist evaluated the images in consensus. Both assessors were blinded to further patient treatment, pathogen detection status or COVID-19 status. The presence of opacifications or consolidations consistent with pneumonia was determined. Additionally, the imaging pattern was assigned to one of the following categories: bilateral and predominantly peripherally located opacifications/consolidations were classified as typical for a COVID-19 infection; the unilateral presence of predominantly peripherally located opacifications/consolidations with no or minimal signs of infection on the contralateral side was rated as almost typical; and opacifications/consolidations limited to one pulmonary lobe consistent with lobar pneumonia were rated as non-typical for a COVID-19 infection. All changes that could not be clearly assigned to one of the aforementioned categories were rated as indeterminate. All images without any signs of infection were classified as none. Figure 1 shows examples of the distribution patterns described (Figure 1).

All CRs included were anonymized, exported as DICOM data and retrospectively analyzed by the Lunit INSIGHT CXR3 DL algorithm (https://insight.lunit.io (accessed on 17 December 2020)). Note that this program only analyzes anterior–posterior or posterior–anterior projections. The algorithm identifies ten radio-morphological pathologies including pulmonal nodules, pneumothorax, fibrosis, atelectasis, cardiomegaly, calcification, pleural effusion, pneumoperitoneum, mediastinal widening and consolidations/opacifications. For this study, we solely focused on the “consolidation/opacification” score, which was validated for detecting all types of pneumonia causing opacification and consolidation [20]. The retrospective analysis was based on abnormality scores. The scores range from 0 to 100 with a threshold of 15 for discriminating normal (<15) from abnormal (≥15) image patterns. The results of the AI-based assessments were compared with the radiologists´ interpretation as the reference method. The AUROC, sensitivity and specificity were evaluated.

### 2.3. Laboratory Testing

COVID-19 infections were confirmed by repeated oronasal swabs or bronchoalveolar lavage and analyzed by RT-PCR. Other suspected pulmonary pathogens were not regularly confirmed by laboratory testing and are not further distinguished in this study.

### 2.4. Statistical Analysis

Statistical analysis was performed using R version 4.0.3 (https://www.r-project.org/ (accessed on 10 February 2021)). Demographic data are shown as the median and corresponding ranges. Categorical parameters are given in total numbers and percentages. Diagnostic performance of the DL algorithm was analyzed using receiver operating characteristic (ROC) and the corresponding area under the receiver operating curve (AUROC) with the assessment of the radiologists as the reference standard. Sensitivity and specificity as well as positive (PPV) and negative predictive values (NPV) were calculated including the corresponding 95% confidence interval (CI). To compare categorical variables, the Chi-square test was used. A *p*-value ≤ 0.05 was defined as statistically significant.

## 3. Results

### 3.1. Patient Collective

A total of 948 patients with a median age of 73 years (range: 18–99 years) were included in this retrospective study (400 female/548 male). A total of 569 patients (60%) tested positive for COVID-19 infection by RT-PCR (237 female/332 male).

### 3.2. Identification of Pneumonia by Radiologists

**Figure 2:**
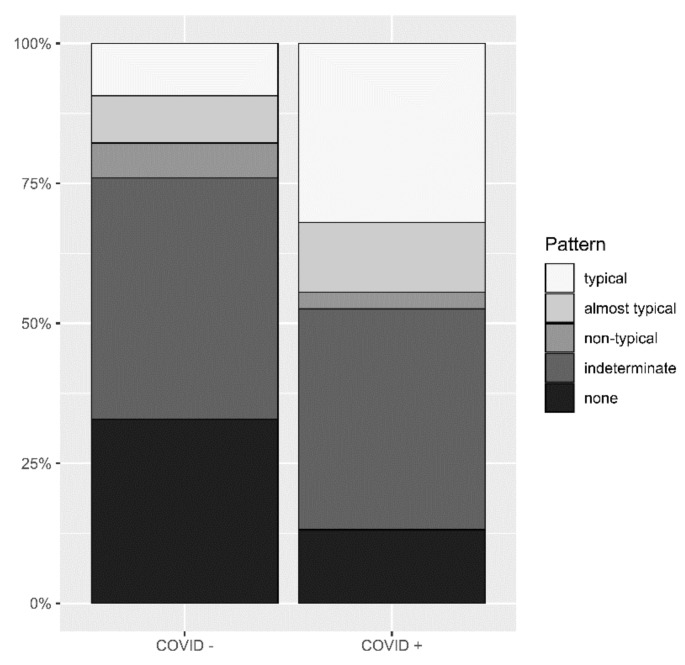
Different distribution patterns in patients with and without COVID-19. Significantly different distribution patterns of opacifications and/or consolidations in chest radiographs between patients with confirmed and ruled-out COVID-19 infections.

Of the 948 CRs performed, radiologists diagnosed consolidations/opacifications consistent with pneumonia in 560 examinations (59.07%). In 388 CRs, no radio-morphological changes for pneumonia were found (40.93%). Because of the ongoing COVID-19 pandemic, the present consolidations/opacifications consistent with pneumonia were classified with regard to a possible COVID-19 infection. The pre-defined and assigned distribution patterns of opacifications/consolidations differed significantly between patients with and without a confirmed COVID-19 infection (*p* < 0.0001) (Figure 2).

Of the 569 confirmed COVID-19 cases within the patient collective, 401 (70.5%) exhibited consolidations/opacifications of varying degrees consistent with a viral pneumonia in their CRs. Among these patients, a typical distribution pattern for predicting a COVID-19 infection corresponds to a sensitivity of 35.9% (CI: 31.2–40.6) and a specificity of 86.8% (CI: 81.5–92.1) with a PPV of 87.3% (CI: 82.8–92.4) and NPV of 34.9% (CI: 30.2–39.6). If the category almost typical is included for predicting a COVID-19 infection, the sensitivity increases to 50.5% (CI: 45.7–55.5), while specificity decreases to 73.0% (CI: 66.1–79.9) with a PPV of 82.5% (CI: 77.8–87.3) and NPV of 36.9% (31.6–42.3).

### 3.3. AI-Based Diagnostic Performance

**Figure 3:**
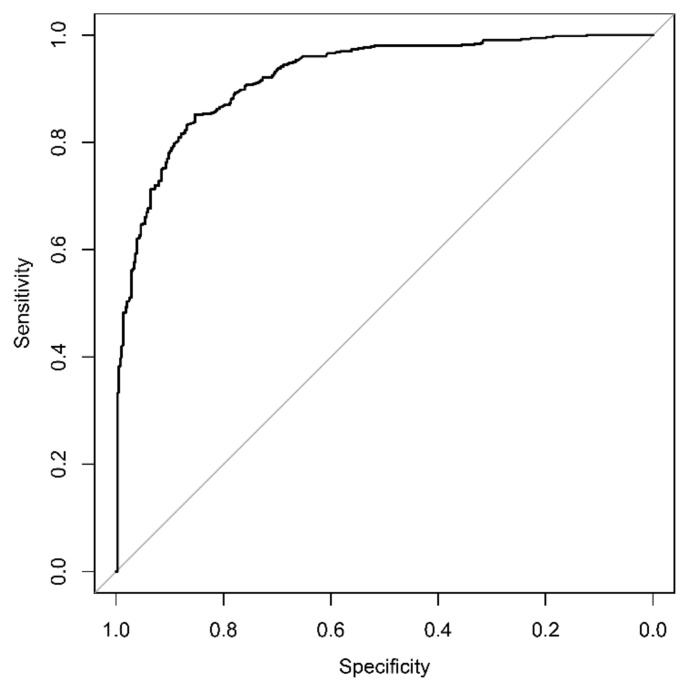
Diagnostic accuracy of the deep learning algorithm for detecting pneumonia. Opacifications and/or consolidations associated with pneumonia can be detected with a corresponding AUROC value of 0.923.

The diagnostic accuracy of the DL algorithm in detecting pneumonia proved to be excellent compared to the radiologists’ assessment, with an AUROC value of 0.923 (Figure 3). For the predefined consolidation threshold value of 15, the sensitivity in detecting pneumonia was 95.4% (CI: 93.6–97.1) with a specificity of 66.0% (CI: 61.3–70.7), a PPV of 80.2% (77.2–83.2) and an NPV of 90.8% (87.4–94.2).

**Figure 4:**
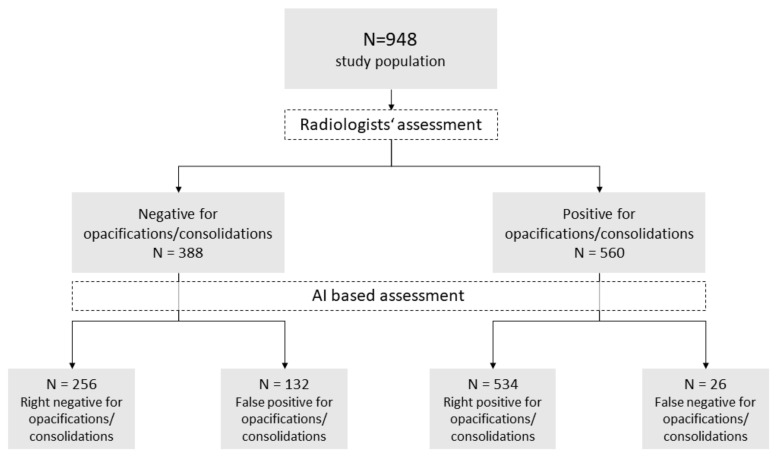
Performance of the artificial intelligence in detecting pneumonia in chest radiographs. Performance of the artificial intelligence in detecting pneumonia in chest radiographs with the radiologists’ assessment as reference standard.

The AI-based evaluation was able to detect 534 out of 560 cases (95.4%) with pneumonia. In 132 CRs, the DL algorithm detected opacifications/consolidations, whereas radiologists did not. During a reassessment by both radiologists, the absence of signs of infection was reconfirmed. In most cases, no abnormality could be detected (see Figure 4).

**Figure 5:**
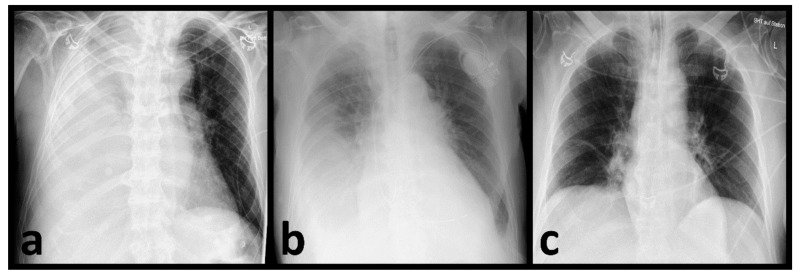
False positive assignments by the AI. Examples of chest radiographs with false positive assignments by the artificial intelligence possibly caused by large pleural effusions (**a**,**b**) or a small dystelectasis in the right lower field (**c**).

However, in some cases, dystelectasis or large pleural effusions might have been misinterpreted by the algorithm. Figure 5 shows examples of CR that have been misinterpreted by the DL algorithm (see Figure 5). Shifting the recommended threshold from 15 to a higher value did not lead to a better evaluation. In 26 CR, AI detected no opacifications, whereas radiologists identified imaging patterns consistent with pneumonia.

## 4. Discussion

In the context of the current pandemic, the role of imaging in rapid and accurate diagnosis has become enormously important. It has been shown several times that different imaging modalities can make a valuable contribution in this regard, whether in the assessment of acute or chronic pulmonary changes [21,22,23]. With this study, we demonstrated that the commercially available DL algorithm used was able to identify consolidations/opacifications associated with pneumonia on plain CR with a very high sensitivity (95.4%) and in the context of an automated pre-evaluation with an absolutely acceptable specificity of 66%, a PPV of 80.2% and an NPV of 90.8%. The algorithm worked robustly in this large real-world patient collective, regardless of the kind of pneumonia. In addition, with respect to the ongoing COVID-19 pandemic, radiologists were able to identify radio-morphological imaging features consistent with a COVID-19 infection based on CR reaching a sensitivity of 50.5% and a specificity of 73%.

Despite the superior accuracy of pulmonary CT, CRs are one of the most important imaging modalities in emergency departments worldwide due to their feasibility. Accelerating the reporting of CRs with relevant abnormalities is of high relevance and can lead to faster treatment and, if necessary, the isolation of the patient. In CRs, consolidations and opacifications are assumed to be mainly caused by infectious diseases [24,25]. Yet, the interpretation of CR, especially in regard to pneumonia, is subject to high variability even among radiologists [8,9]. This issue is even more prevalent where CRs are performed by clinicians in the absence of radiologists. Studies have shown that relevant misinterpretations of pneumonic patterns in CRs occur between the interpretations of CR in radiologists and clinicians [26,27]. Accordingly, the additional evaluation and highlighting of findings in CRs by AI is advisable. Recent studies have proved that with the help of DL algorithms the diagnosis of pneumonia (among others, including COVID-19 infections) can be detected with high sensitivity in CR [10,14,18,28]. Tajmir et al. even showed that with the help of AI, the inter-observer variability in the interpretation of imaging can be reduced [29]. Another advantage of using AI compared to human-based analysis is the non-existence of fatigue, leading to the risk of missing significant abnormalities in CRs [30]. In this respect, a robust DL algorithm supports rapid and reliable reporting. An integrated AI tool could highlight suspicious patterns, increase radiologists’ and clinicians’ awareness of suspicious pulmonary patterns and lead to more objective diagnoses.

In 132 patients, the AI falsely assessed the presence of pneumonia. However, in some of these patients, large pleural effusions were found, which may also require urgent treatment. In the study by Jang et al., false positive interpretations by the DL algorithm were attributed to increased vascular marking, emphysematous changes, interstitial thickening and subsegmental atelectasis [11].

All 26 falsely negative classified cases for signs of pneumonia by the DL algorithm showed slight radio-morphological pathologies according to the radiologists. Radiologists’ classifying of these mild changes as pneumonia may be attributable to the high prevalence of COVID-19 infections during the pandemic and the respiratory symptoms of the patients. This represents a human bias to which the AI is not subject. The commercially available product used in this study is the Lunit INSIGHT CXR3. It comprises ten radio-morphological patterns including pulmonal nodules, pneumothorax, fibrosis, atelectasis, cardiomegaly, calcification, pleural effusion, pneumoperitoneum, mediastinal widening and consolidations/opacifications. Other studies have proved that this program’s performance in detecting the aforementioned imaging patterns is excellent [11,31,32]. With the recommended cut-off value of 15 for the consolidation score, a high sensitivity (95.4%) and moderate specificity (66.0%) were achieved, which is optimal for application in the context of an automated preliminary assessment. Therefore, the applied cut-off value should identify all patients with CR as suspicious for consolidations/opacifications, even in a post-pandemic scenario. The high accuracy of AI-based analyses of opacifications/consolidations in CR is important during pre-testing for the prioritization of further assessments. During our evaluation, AI showed an excellent AUROC value of 0.923. This value is similar to the published AUROC value of 0.921 by Jang et al. They evaluated the accuracy of AI on 279 COVID-19-positive individuals [11]. We can conclude, therefore, that the DL algorithm works robustly. Jang et al. reported comparable sensitivity (95.6%) but a significantly higher specificity of 88.7%. This difference is explained by the fact that only patients with confirmed COVID-19 infections were included, in contrast to the representative real-world population including all patients with respiratory symptoms used in this study. Therefore, the Lunit INSIGHT CXR3-based analysis of opacifications/consolidations is not limited to detecting COVID-19 infections and has been applied in real-life scenarios without any prior patient selection.

As the COVID-19 pandemic is still prevalent, we controlled the radio-morphological changes regarding COVID-19 pneumonia.

The classification of radio-morphological findings into ‘typical’, ‘non-typical’ or ‘indeterminate’ for COVID-19 infection has been widely applied during the COVID-19 pandemic. When unilateral peripheral opacifications/consolidations were added to the ‘typical’ class, COVID-19 infections were identified with a sensitivity of 50.5%, a specificity of 73.0%, a PPV of 82.5% and an NPV of 36.9%. This is important, since unilateral morphological changes as a distribution pattern have so far only been included in a few studies, although they are also described as a typical pattern for COVID-19 infection [33,34].

The sensitivity for correctly detecting COVID-19 infections in CR in our study appears low (50.5%). Cozzi et al. achieved a sensitivity of 89–92.8% in their real-world analysis during the pandemic [35]. However, their study reports a low to moderate specificity of 40.7–66.0%, while we achieved a specificity of 86.8%.

In the ongoing pandemic, the faster detection of pulmonary infections by analysis of CRs may lead to faster isolation of patients and therefore might reduce the risk of spreading the infection. Nevertheless, prospective studies need to follow with a focus on the practicability of AI use in clinical processes and integration into clinical decision making [36]. This is of special relevance as some studies showed ambivalent results when combining AI with clinical processes [28,37]. A beneficial effect on the workflow and turnaround times is not readily assured. However, the combined reporting of images by AI and radiologists is a promising model. The high sensitivity of AI can be complemented by the comparatively high specificities of radiologists. Thus, the best possible assessment of a CR could be achieved.

The main limitation of this analysis is its retrospective character. The achievable time benefit is therefore theoretical and largely dependent on the way the software is integrated into existing infrastructure. However, the used AI software is commercially available and can usually be put into operation within two weeks. By blinding the radiologists to the further treatment of the patients or the results of COVID-19 tests, a realistic scenario for interpreting CR in the emergency department was simulated. However, in comparison to the AI, radiologists still have the advantage of including more information about the patient in their interpretation, such as blood parameters, symptoms and known comorbidities. Although some programs already have the functionality to take up this information, their use is not yet widespread [38]. This study did not investigate the possible integration of AI into clinical routines, but that integration is a very important question and should be the subject of future studies. Another factor limiting the applicability of the results to other populations is the comparatively high prevalence of pneumonic infections during the pandemic, which could have biased the results. Thus, it would be more accurate to speak of a real-pandemic collective rather than a real-world one.

## 5. Conclusions

Based on the currently largest-available patient collective assessed during the COVID-19 pandemic, this study demonstrates the high sensitivity (95.4%) and acceptable specificity (66%) of a DL algorithm in detecting pneumonia in CRs. The corresponding AUROC value reached an excellent value of 0.923, underlining its robustness, which was not necessarily guaranteed in advance. In addition, based on the evaluation of specific patterns of findings by experienced radiologists, the diagnosis of COVID-19 pneumonia was achieved with a sensitivity of 50.5% and a comparatively high specificity of 73%. The combination of the algorithm’s high sensitivity with the radiologists’ high specificity seems to be of great value. The pre-selection and highlighting of suspicious CR by AI could hasten the reporting of pulmonal infections and therefore may shorten the turnaround time, aiding faster clinical decision-making. However, the integration of AI into everyday clinical practice remains an open challenge and is still subject to further investigation.

## Data Availability

Not applicable.
